# Comparison of Conventional DWI, Intravoxel Incoherent Motion Imaging, and Diffusion Kurtosis Imaging in Differentiating Lung Lesions

**DOI:** 10.3389/fonc.2021.815967

**Published:** 2022-01-20

**Authors:** Yu Zheng, Jie Li, Kang Chen, Xiaochun Zhang, Huan Sun, Shujiao Li, Xie Zhang, Zhenping Deng, Na Liang, Shihong Li

**Affiliations:** ^1^ Department of Radiology, Chengdu Second People’s Hospital, Chengdu, China; ^2^ Department of Radiology, Huadong Hospital Affiliated With Fudan University, Shanghai, China

**Keywords:** apparent diffusion coefficient, intravoxel incoherent motion, diffusion kurtosis imaging, magnetic resonance imaging, lung lesions

## Abstract

**Purpose:**

To compare conventional diffusion weighted imaging (DWI), intravoxel incoherent motion imaging (IVIM) and diffusion kurtosis imaging (DKI) in differentiating malignant and benign lung lesions.

**Method:**

Fifty-five consecutive patients with lung lesions underwent multiple b-value DWI. The apparent diffusion coefficient (ADC), IVIM and DKI parameters were calculated using postprocessing software and compared between the malignant and benign groups. Receiver operating characteristic (ROC) analysis was performed for all parameters.

**Results:**

ADC and D were lower in malignant lesions than in benign lesions, while Kapp was higher (*P* < 0.05). The differences in D*, f, and Dapp between the two groups were not significant (*P* > 0.05). The areas under the curves (AUCs) of ADC, D, and Kapp were 0.816, 0.864, and 0.822. The combination of all the significant parameters yielded an AUC of 0.880. There were no significant differences in diagnostic efficacy among ADC, D, Kapp and the predictor factor (PRE).

**Conclusions:**

In this study, traditional DWI (ADC), IVIM (D), and DKI (Kapp) all had good diagnostic performance in differentiating malignant lung lesions from benign lesions, but the combination of ADC, D, and Kapp value had better diagnostic efficacy than these parameters alone.

## Introduction

Lung cancer is the leading malignant tumour in the world in terms of morbidity and mortality (1). The accurate diagnosis and differential diagnosis of patients with lung lesions are beneficial to the selection of treatment options. Because of the overlap of the morphological characteristics of benign and malignant lesions, it is challenging to distinguish them on computed tomography (CT) based on morphology. Positron emission tomography (PET)-CT is not only expensive but also has a high false-positive rate. Some inflammatory lesions also have high fluorodeoxyglucose (FDG) uptake (2).

In recent years, with the development of magnetic resonance (MR) technology, traditional diffusion-weighted imaging (DWI) has become a means to distinguish benign and malignant lung lesions (3). However, the apparent diffusion coefficient (ADC), which reflects the degree of diffusion limitation, not only reflects the diffusion of water molecules in tissues but is also affected by microcirculation (4). Compared with conventional DWI, intravoxel incoherent motion (IVIM) can separate the diffusion of water molecules and the microcirculation of tissues (5). Theoretically, IVIM can more accurately reflect the diffusion of water molecules in tissues. IVIM has been proven useful for the identification of tumours (6). Currently, the diagnostic performance outcomes of IVIM parameters in the differentiation of lung lesions are inconsistent (7).

In addition, diffusion kurtosis imaging (DKI) can reflect the diffusion of water molecules in a non-Gaussian distribution and the complexity of tissues (8). At present, DKI is mainly used in the study of the central nervous system, abdomen, pelvis, breast and other body parts, while it is less commonly used in the study of pulmonary lesions (4, 9).

Therefore, the purpose of this study was to compare traditional DWI, IVIM and DKI in differentiating benign and malignant lung lesions, providing more references for clinical practice.

## Materials and Methods

### Patients

This prospective study was approved by the local ethics committee. Written informed consent was obtained from all patients. Sixty-five patients with lung lesions from October 2019 to November 2020 were enrolled.

The inclusion criteria were as follows (1): All patients had pulmonary lesions found by CT, the diameter of the lesions was ≥ 10 mm (3), and the solid component was more than 50% (2). Pathological results were obtained, and some inflammatory lesions were confirmed by follow-up after anti-inflammatory treatment (3). All patients underwent an MRI scan within 1 week after lung lesions were found on CT and did not undergo any treatment before scanning.

The exclusion criteria were as follows (1): contraindications of MRI scanning; and (2) unsatisfactory imaging quality.

### Image Acquisition

All MRI examinations were performed with a 3.0T MR scanner (MAGNETOM Prisma, Siemens Healthcare, Erlangen, Germany) using a 32-channel body coil and an integrated spine coil. Routine scanning included T1-volumetric interpolated breath-hold examination (VIBE) and coronal and transverse half-Fourier-acquisition single-shot turbo spin-echo (HASTE) T2-weighted imaging (T2WI). The following parameters were employed for conventional axial T2WI: TR/TE, 1400/91 ms; slice thickness, 5 mm; intersection gap, 0.3 mm; matrix, 320×320; and FOV, 400 mm×400 mm.

A multiple b-value DWI with a single-shot echo-planar imaging pulse sequence in the axial orientation during free breathing was performed. The parameters were as follows: twelve b values from 0 to 2000 s/mm^2^ (b=0, 20, 60, 80, 150, 200, 400, 600, 800, 1200, 1600, and 2000 s/mm^2^); TR/TE, 4000/54 ms; slice thickness, 5 mm; gap, 0.2 mm; matrix, 128×128; FOV, 380 mm×380 mm; directions, 3; and scanning time, 8 min 24 s (**Table 1**).

**Table 1:** Scanning parameters of MRI.

Sequence	T2WI-Haste	T1-VIBE	Multiple b-value DWI
TR/TE(ms)	1400/90	3.97/1.23	4000/54
Slice thickness(mm)	5	3	5
Intersection gap(mm)	0.3	0.2	0.2
Matrix	320×195	320×195	128×84
FOV (mm)	400×400	440×440	380×380
Directions	–	–	3
Scanning time	40s	15s	8min24s

### Image Data Analysis

The MRI data were processed using Whole Body Diffusion Toolbox software (Siemens Medical Systems). Multiple b-value DWI data were postprocessed with different models. ADC was calculated using the monoexponential model from DWI with b values of 0 and 800 s/mm² using a monoexponential fit of signal intensity with the following equation (4):


$${\text{S}}_{\text{b}}{\text{/S}}_{0}=\exp(-\text{b}\times \text{ADC}),$$

where S_b_ represents the signal intensity at a specified b value, and S_0_ is the signal intensity at b = 0 s/mm^2^.

Meanwhile, the IVIM parameters (D, D*, and f) were obtained with biexponential fit models using 9 b values (0, 20, 60, 80, 150, 200, 400, 600, and 800 s/mm^2^). The DWI signal intensity and b factors were fitted to the following equation (5):


$${\text{S}}_{\text{b}}{\text{/S}}_{0}=[(1-\text{f})\exp(-\text{b}.\text{ D})+\text{f}.\exp)-\text{b}(\text{D}+\text{D}*)]$$

where D is the true diffusion coefficient representing pure molecular diffusion, D* is the pseudo-diffusion coefficient representing incoherent microcirculation, and f is the fraction of perfusion.

In addition, DKI parameters (Kapp and Dapp) were derived using six b value signal intensities (b = 0, 600, 800, 1200, 1600, and 2000 s/mm²). The following equation was used for the DKI parameter calculation (10):


$${\text{S}}_{\text{b}}{\text{/S}}_{0}=\exp(-\text{b}.\text{ Dapp}+{\text{b}}^{2}\cdot {\text{Dapp}}^{2}\cdot \text{Kapp/}6)$$

where Kapp is a unitless parameter, representing the deviation of water motion from Gaussian diffusion, and Dapp is the kurtosis-corrected diffusion coefficient.

Two radiologists (with 5 and 8 years of experience in thoracic MRI) who were blinded to the pathologic results drew regions of interest (ROIs) on the ADC maps and recorded the values of each parameter independently. Each lesion was outlined twice, and then the average value was taken for analysis. Tumour ROIs were drawn by outlining tumour borders on ADC maps showing the largest tumour cross-sections and avoiding necrotic areas and adjacent large vessels by referring to T2WI and DWI images. The minimum size of ROIs is 121mm^2^. The ROIs were automatically copied from the ADC maps to the corresponding IVIM and DKI parametric maps to obtain the values of D, D*, f, Dapp, and Kapp.

### Statistical Analysis

The data are presented as median and interquartile range. The intraclass correlation coefficient (ICC) with a 95% confidence interval (CI) was used to evaluate interobserver agreement for parameter measurements (0.00-0.20, poor agreement; 0.21-0.40, fair agreement; 0.41-0.60, moderate agreement; 0.61-0.80, good agreement; and 0.81-1.00, excellent agreement) (11).

ADC, IVIM and DKI parameters were compared between the malignant and benign groups by Student’s t-test or the Mann-Whitney U test. A *P* < 0.05 was considered statistically significant. The significant parameters were fitted by logistic regression, and a predictive factor (PRE) was generated. Receiver operating characteristic (ROC) curve analyses were further performed to evaluate the diagnostic efficacy and determine the optimal cut-off value of each parameter in predicting malignancy. The areas under the curve (AUCs), sensitivity, specificity, and Youden index were calculated. AUCs were compared using the DeLong method (12). Statistical analyses were performed with SPSS 22.0 (IBM SPSS Statistics, USA), GraphPad Prism 5.0 (Prism, USA) and MedCalc 19.0.4 (MedCalc, Ostend, Belgium).

## Results

### Patients’ Characteristics

A total of sixty-five patients were consecutively included in our study. Four patients were excluded due to MRI contraindications, and six patients were excluded due to inferior imaging quality. The remaining fifty-five patients were enrolled in the final cohort, including twenty-nine males (52.73%) and twenty-six females (47.27%) with an average age of 54 years (range of 32–86 years). Among the fifty-five patients included, thirty-two patients had malignant tumours and twenty-three had benign lesions. Malignant lesions were adenocarcinoma (n=19), squamous cell carcinoma (n=6), large cell carcinoma (n=2), small cell lung cancer (SCLC) (n=4) and solitary metastatic tumour (n=1). Benign lesions were pulmonary tuberculosis (n=10), organizing pneumonia (n=7), inflammatory pseudotumour (n=5), and sclerosing pneumocytoma (n=1).

### Comparison of ADC, IVIM and DKI Parameters Between Malignant and Benign Lesions

The interobserver reproducibility ranged from good to excellent for the ADC, IVIM and DKI parameters (ADC: ICC = 0.848, 95% CI = 0.753–0.909; D: ICC = 0.881, 95% CI = 0.804–0.929; D*: ICC = 0.810, 95% CI = 0.695–0.885; f: ICC = 0.706, 95% CI = 0.544–0.817; Kapp: ICC = 0.845, 95% CI = 0.748–0.907; Dapp: ICC = 0.811, 95% CI = 0.697–0.885).

**Figure 1:**
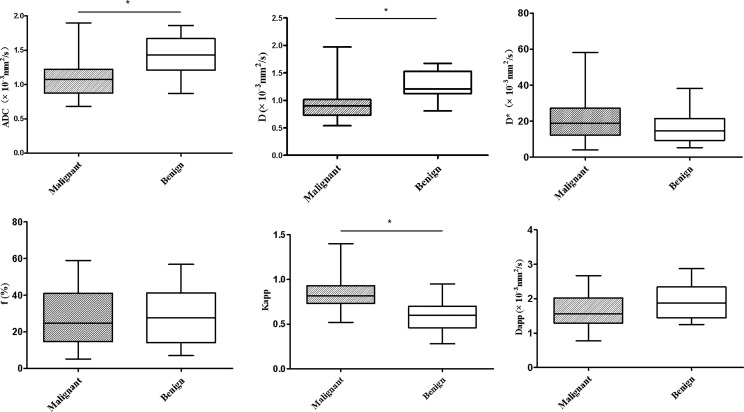
Whiskers boxplots of ADC, D, D*, f, Kapp, Dapp values of malignant and benign lung lesions. *p < 0.05.

**Figure 2:**
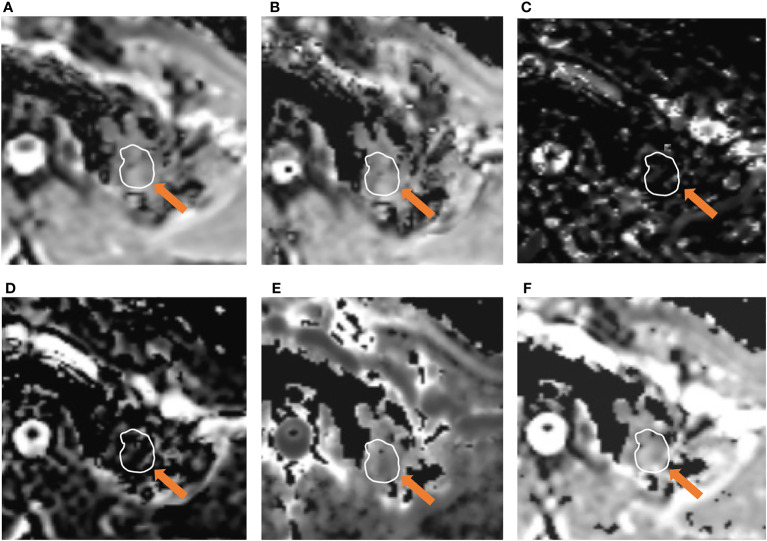
A 51-year-old male was diagnosed with pulmonary tuberculosis in the left upper lobe. Parameter map of ADC, D, D*, f, Kapp, and Dapp calculated from multiple b-value DWI data **(A–F)**. The calculated mean values of ADC, D, D*, f, Kapp, and Dapp were 1.21×10^-3^ mm^2^/s, 1.13×10^-3^mm^2^/s, 8.74×10^-3^ mm^2^/s, 12.62%, 0.65, and 2.34×10^-3^ mm^2^/s, respectively.

**Figure 3:**
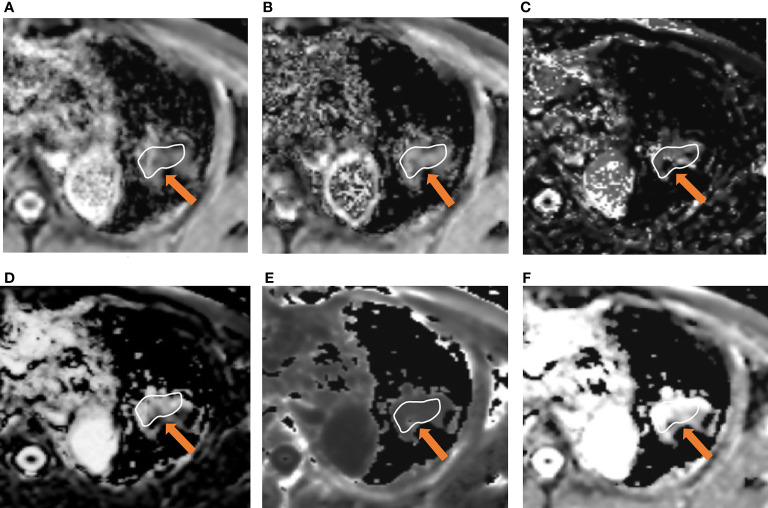
A 66-year-old male was diagnosed with lung adenocarcinoma in the left upper lobe. Parameter map of ADC, D, D*, f, Kapp, and Dapp calculated from multiple b-value DWI data **(A–F)**. The calculated mean values of ADC, D, D*, f, Kapp, and Dapp were 0.84×10^-3^ mm^2^/s, 0.65×10^-3^mm^2^/s, 35.23×10^-3^ mm^2^/s, 32.31%, 1.01, and 2.21×10^-3^ mm^2^/s, respectively.

The differences in ADC, IVIM and DKI parameters between benign and malignant lesions are shown in **Table 2** and **Figure 1**. The ADC and D values of malignant tumours were lower than those of benign lesions, while the Kapp value of malignant tumours was higher than that of benign lesions, and the differences were statistically significant (*P* < 0.05). The differences in D*, f, and Dapp between these two groups were not significantly different (*P*> 0.05). Typical cases are shown in **Figures 2** and **3**.

**Table 2:** The differences of ADC, IVIM and DKI parameters between malignant and benign lesions.

Parameters	Malignant (n = 32)	Benign (n = 23)	*P*
ADC(×10^-3^mm^2^/s)	1.075 (0.875, 1.22)	1.43 (1.21, 1.67)	0.000
D(×10^-3^mm^2^/s)	0.90 (0.73, 1.015)	1.21 (1.12, 1.53)	0.000
D^*^(×10^-3^mm^2^/s)	18.725 (12.263, 27.18)	14.63 (9.29, 21.41)	0.138
f (%)	24.65 (14.615, 40.97)	27.56 (14.07, 41.23)	0.675
Kapp	0.81 (0.67, 0.90)	0.66 (0.575, 0.71)	0.002
Dapp(×10^-3^mm/s)	1.56 (1.293, 2.018)	1.87 (1.44, 2.34)	0.079

### Diagnostic Efficiency of Quantitative Parameters

**Figure 4:**
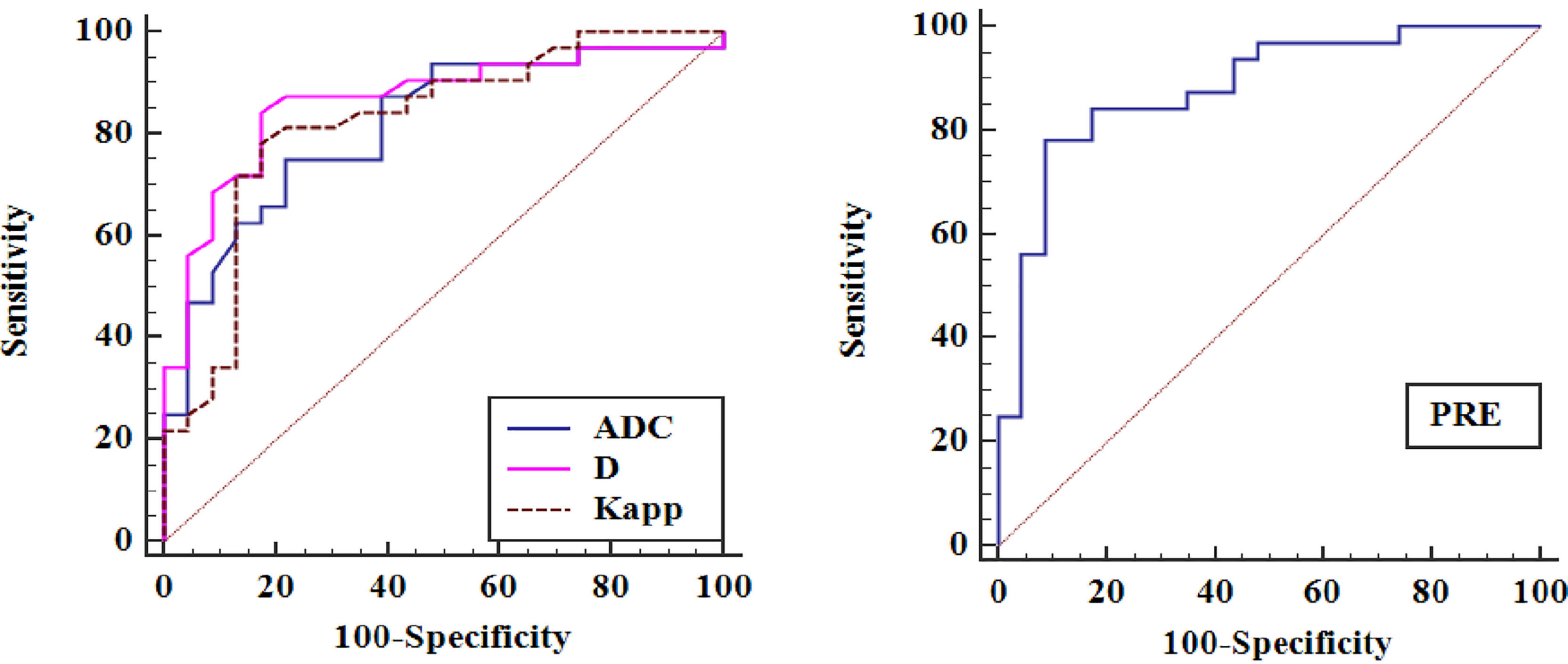
Receiver operating characteristic curves (ROCs) for ADC, D, Kapp and PRE in distinguishing pulmonary malignant tumours from benign lesions.

The ROC curves of quantitative parameters for distinguishing malignant lung lesions from benign lesions are shown in **Figure 4**, and the corresponding diagnostic performance are plotted in **Table 3**. The AUCs of ADC, D, and Kapp were 0.816, 0.864, and 0.822, respectively. Among the significant parameters, the best predictive parameter was D (AUC = 0.845, cut-off value ≤ 1.07 × 10^-3^ mm^2^/s, sensitivity = 84.37%, and specificity = 82.61%). The above factors were then fitted by logistic regression, and a PRE was generated, the results of logistic regression are shown in **Table 4**. The PRE achieved a high AUC of 0.887, with 91.3% sensitivity and 78.12% specificity. There were no statistically significant differences in diagnostic efficacy among ADC, D, Kapp and PRE when pairwise comparisons of ROC curves were performed.

**Table 3:** Diagnostic efficiency of quantitative parameters.

Parameters	Cut-offvalue	Sensitivity (%)	Specificity (%)	AUC (CI95%)	Youden index	*P*
ADC(×10^-3^mm^2^/s)	≤1.19	75.00	78.26	0.816 (0.688-0.908)	0.533	<0.0001
D(×10^-3^mm^2^/s)	≤1.07	84.37	82.61	0.864 (0.745-0.942)	0.670	<0.0001
Kapp	>0.72	78.12	82.61	0.822 (0.695-0.912)	0.607	<0.0001
PRE	≤0.328	91.30	78.12	0.880 (0.765-0.952)	0.694	<0.0001

**Table 4:** Regression analysis of ADC, D, and Kapp value.

Parameters	Regression coefficient	Standard error	Wald	*P*
ADC(×10^-3^mm^2^/s)	-4.528	1.363	11.071	0.001
D(×10^-3^mm^2^/s)	-5.064	1.472	11.83	0.001
Kapp	5.606	2.031	7.616	0.006

## Discussion

We found that the DWI-derived parameter ADC, the IVIM-derived parameter diffusion coefficient D and the DKI-derived parameter diffusion kurtosis Kapp are useful for the diagnosis of lung lesions. Compared with benign lesions, malignant lesions had significantly lower D and ADC values and significantly higher Kapp values. Among these significant parameters, the D value had a higher AUC than the ADC and Kapp values. Finally, the combination of all the significant parameters fitted by logistic regression yielded an AUC of 0.880, which had the strongest predictive value. Therefore, the combination of ADC, D, and Kapp value had better diagnostic efficacy than these parameters alone

DWI, characterizing the restriction of random thermal motion of water molecules, has been applied for the detection and characterization of lung lesions. Several studies (13–16) have shown that the ADC value of malignant lung tumours is significantly lower than that of benign lesions. Our data showed that the average ADC values were higher than their D values. The reason may be that ADC not only reflects the diffusion of water molecules but is also affected by microcirculation perfusion. In this study, DWI with multiple b values (b values from 0 to 2000 s/mm^2^) were used, and it was found that the ADC and D values of malignant lung lesions were significantly lower than those of benign lung lesions. The reason may be that malignant tumour cells proliferate rapidly, resulting in high cell density. In addition, malignant tumour cells have larger nuclei and less cytoplasm, which reduces the extracellular space and aggravates the restriction of water molecules.

However, the diffusion parameter Dapp derived from DKI showed a lower trend in malignant tumours, but there was no significant difference between the two groups, which was inconsistent with the findings of some previous studies (9, 17, 18), which found that the Dapp of malignant lesions was significantly lower than that of benign lesions. This discrepancy may be due to the heterogeneity of the tumour and the size and number of b values selected in DKI. In addition, the Kapp of malignant lung tumours was significantly higher than that of benign lung lesions. Theoretically, the cell structure and tissue environment of malignant tumours are more complex than those of benign lesions, resulting in a more obvious non-Gaussian distribution of tumour tissues and a higher diffusion kurtosis value. Previous studies have also shown that the diffusion kurtosis of malignant tumours is higher than that of benign lesions (17–19).

Our study showed that the D value has better diagnostic efficiency than the ADC value, which may be related to its imaging principles because it only reflects the pure diffusion of water molecules without the influence of microcirculation perfusion and can more accurately reflect the restriction of tissue water molecules. In addition, the combination of all the significant parameters fitted by logistic regression yielded an AUC of 0.887, which shows that compared with a single indicator, the combined indicators can more accurately distinguish malignant and benign lung lesions in clinical practice.

Some previous studies have shown that f is significantly meaningful in the diagnosis of lung lesions (20, 21) and other lesions (22, 23). In this study, the perfusion-related parameters D* and f were not significantly different between malignant lung tumours and benign lesions, which is in line with the findings of previous studies (4, 24, 25). The reason may be that the repeatability of D* and f values in pulmonary diffusion imaging is poor and has great variability, which is affected by the shape, size and location of the lesion (26). Similarly, in our study, the ICCs for f were lower than those of the other parameters. Moreover, the f value is affected by the T2 contributions of both perfusion and pure molecular diffusion compartments (11).

This study has some limitations. First, the sample size was not large, and the types of lung lesions were limited; therefore, it is necessary to expand the sample size and increase the types of pulmonary lesions in further research. Second, there is no unified standard for the selection of b values in IVIM and DKI imaging. Although more b values can improve the accuracy, the corresponding scanning time will be prolonged. Third, the mean value of the ROI was taken to define the measured parameter, and perhaps a histogram and volumetric analysis of these heterogeneous lesions may have been more useful to diagnose these lesions.

In conclusion, our results indicate that mean ADC, D, and Kapp values are useful for the discrimination between pulmonary malignant and benign lesions, which indicates that multiple b-value DWI provides additional information for clinical diagnosis. Larger, prospective studies are needed to confirm our findings.

## Data Availability Statement

The original contributions presented in the study are included in the article/supplementary material. Further inquiries can be directed to the corresponding authors.

## Ethics Statement

The studies involving human participants were reviewed and approved by the Ethic Committee of the Huadong Hospital Affiliated with Fudan University. The patients/participants provided their written informed consent to participate in this study.

## Author Contributions

NL and SHL conceived and presented idea. JL and YZ collected the data. KC, XCZ, HS, SJL, XZ and ZD analyzed the data. YZ drafted the manuscript. All authors reviewed the manuscript, and NL made corrections to the manuscript. All authors contributed to the article and approved the submitted version.

## Funding

This study was supported by Medical Scientific Research Project of Sichuan Province (S17007), and the General Project from Shanghai Municipal Health Bureau (CN) (201940174).

## Conflict of Interest

The authors declare that the research was conducted in the absence of any commercial or financial relationships that could be construed as a potential conflict of interest.

## Publisher’s Note

All claims expressed in this article are solely those of the authors and do not necessarily represent those of their affiliated organizations, or those of the publisher, the editors and the reviewers. Any product that may be evaluated in this article, or claim that may be made by its manufacturer, is not guaranteed or endorsed by the publisher.

## Abbreviations

ROIs, regions of interest; ICC, intraclass correlation coefficient; CI, confidence interval; PRE, predictive factor; AUCs, areas under the curve.
